# From the President’s Desk: Part 1, 2025

**DOI:** 10.4102/safp.v67i1.6099

**Published:** 2025-02-11

**Authors:** Tasleem Ras

**Affiliations:** 1 Division of Family Medicine, Department of Family, Community and Emergency Care, Faculty of Health Sciences, University of Cape Town, Cape Town, South Africa; 2 South African Academy of Family Physicians, Durbanville, South Africa

Dear colleagues,

I have the great honour to pen this communication as the President of the SA Academy of Family Physicians. Over the last few decades, the academy has played a leading role in advocating and organising for the establishment of the discipline of family medicine and the enhancement of district and primary care services in our country. The next 5 years see us building on this work, with several interesting challenges and opportunities before us. The extent to which we respond will largely be dependent on your active engagement in the academy’s structures and activities.

One of the most significant developments of 2024 has been the unification, under the banner of the academy, of all organisations representing specialist family physicians in private practice in the country. A position article on the contributions of family physicians in the private sector was published (https://doi.org/10.4102/safp.v66i1.6022), and in December, a decision was made to include a private practice representative in the Academy Council. Furthermore, Dr Sheena Matthew, also in private practice, was elected Vice President of the Academy. These developments expand the potential of our organisation, networks, and work opportunities for emerging family physicians.

A second very exciting development is in our conference domain. As you may already know, the academy was awarded host status for the World WONCA Congress (2027), with Cape Town being the chosen venue. We have already secured the Cape Town International Convention Centre, booked hotel rooms, and made formal connections with the Wesgro, the official tourism agency for the Western Cape. Moreover, for the 2026 congress that will be held in Gauteng, we are in discussions to host the first ever interdisciplinary primary care conference, hoping to bring together a diverse group of professionals representing the multidisciplinary nature of healthcare. The 2025 congress will be jointly hosted in Tshwane by Sefako Makgatho and Free State Universities. We look forward to seeing you there!

Planetary sustainability is an important guiding principle to the academy. To this end, we have a standing commitment to ensure that all our activities have as small a carbon footprint as possible and consistently donate to a planetary health organisation from our conference earnings.

Financial sustainability is also a priority, and the Council has identified some avenues to securing this. Chief among these strategies is enhanced membership, including registrar and associate membership, as member fees fund the daily operational demands of running a national organisation. We have also established a mechanism for the issuing of Section 18A certificates, which secures tax rebates for anyone wishing to make a monetary contribution to the academy. We are in the process of negotiating with private entities that would see the academy earn royalties from certain public activities and are actively pursuing training grant opportunities. The current council will be working hard towards long-term financial stability over the next 5-year period.

It would be remiss not to mention that we have established a registrar-driven social media committee in 2024 that has seen modest successes in enhancing our social media profile. In 2025, we intend to strengthen this development, and welcome all our members to like, subscribe and comment on our posts.

There are many more activities and achievements that could be mentioned, and I urge all members to engage with our social media platforms, to ensure that you are kept up to date with all the latest developments. Our collective actions and solidarity will ensure that we grow together, in the spirit of Ubuntu, building on the rich legacies of those who have come before us.

Wishing all of us a prosperous and productive 2025.

